# Comparison of Two Methodologies for CD4+ T Lymphocytes Relative Counting on Immune Monitoring of Patients with Human Immunodeficiency Virus

**DOI:** 10.1100/2012/906873

**Published:** 2012-11-28

**Authors:** Danielle Cristyane Kalva Borato, Emerson Carraro, Sônia Regina Weber Ribas, Carlos Augusto Kalva-Filho, José Carlos Rebuglio Vellosa

**Affiliations:** ^1^Pharmaceutical Sciences Post Graduate Program, State University of Ponta Grossa (UEPG), General Carlos Cavalcanti Avenue, 4748 Uvaranas, 84030-900 Ponta Grossa, PR, Brazil; ^2^Department of Pharmacy, State University in the Paraná Midwestern (UNICENTRO), CEDETEG Campus, 85040-080 Guarapuava, PR, Brazil; ^3^County Health Department, Expert Assistance Service (EAS), City Hall, 84051-900 Ponta Grossa, PR, Brazil; ^4^Department of Physiotherapy, State University Paulista (UNESP), Presidente Prudente Campus, 19060-900 Presidente Prudente, SP, Brazil

## Abstract

Considering that counting the percentage of CD4 T lymphocytes can add prognostic information regarding patients infected with HIV, the aim of this study was to evaluate the percentage values of CD4+ T lymphocytes from 81 patients determined by flow cytometry and estimated by flow cytometry in conjunction with a hematology counter. Means were compared through the Student's *t*-test. Pearson's correlation was determined, and the agreement between results was tested by Bland-Altman. The level of significance was *P* < 0.05. It was found a significantly higher mean difference between the relative values of CD4+ T lymphocytes to the hematologic counter (*P* < 0.05), for all strata studied. Positive and significant correlations (*P* < 0.01) were found between the strata CD4 < 200 cells/mL (*r* = 0.93), between 200 and 500 cells/mL (*r* = 0.65), and >500 cells/mL (*r* = 0.81). The limits of agreement were 1.0 ± 3.8% for the stratum of CD4 < 200 cells/mL, approximately 2.2 ± 13.5% for the stratum of CD4 between 200 and 500 cells/mL, and approximately 6.2 ± 20.4% for the stratum > 500 cells/mL. The differences in the percentages of CD4+ T lymphocytes obtained by different methodologies could lead to conflict when used in clinical decisions related to the treatment and care of people infected with HIV.

## 1. Introduction

The natural history of infection with the human immunodeficiency virus (HIV) is characterized by a progressive decline of T helper (CD4+) lymphocytes [1]. This depletion occurs because the virus infects and kills CD4+ T lymphocytes, the main mechanism for programmed cell death apoptosis [2]. These cells act as regulators and amplifiers of the immune response and are associated with the immunopathogenesis of HIV infection. Thus, the decline of CD4+ T cells results in an impaired immune system and the progression of infection (the main consequence of the onset of opportunistic infections) to AIDS (human immunodeficiency syndrome) and death due to conditions associated with infection [3].

The level of CD4+ T cells is considered to be one of the most important immunological parameters in HIV-infected individuals to evaluate their prognosis and state of immune deficiency, to determine the start of antiretroviral therapy, to monitor the effectiveness of this treatment, to evaluate the need to start or discontinue prophylaxis for opportunist infections [4], and to establish the diagnosis of AIDS [5]. 

Thus, quantification of CD4+ lymphocytes (immunophenotyping by flow cytometry) is an indispensable procedure in the evaluation of patients with HIV [6]. Immunophenotyping provides important information about the leukocytes of the immune system, distinguishing total lymphocytes (CD45+), T lymphocytes (CD3+), and subtypes of T lymphocytes which comprise two subsets: helper T cells (T lymphocytes CD3+/CD4+) and cytotoxic T cells (T lymphocytes CD3+/CD8+) [7]. Thus, the total lymphocyte count and percentage values of lymphocyte subsets may be determined by using flow cytometry, using CD45+ monoclonal antibody, in association with CD3+, CD4+, and CD8+ antibodies [8].

The absolute count of lymphocytes may be influenced by biological factors that affect the total count of leukocytes and lymphocytes, such as the use of drugs that suppress the bone marrow, acute infections (e.g., sepsis, malaria, and tuberculosis), and pregnancy, which can lead to hemodilution [9]. Besides these biological factors, there could also be a variation due to methodological factors such as differences in the methods and equipment used [3, 10, 11].

Several studies have reported that variations in the percentage count of CD4+ T lymphocytes are more stable parameters than variations in the absolute count to assess the progression of the disease [12–15]. Moreover, the relative values of CD4+ T lymphocytes in the initiation of antiretroviral therapy were associated with the risk of disease progression independent of other clinical factors, including absolute counts of CD4+ T lymphocytes [16].

The main concern regarding the use of counting the percentage of CD4+ T cells is how the variation of results could have an influence on decisions related to the clinical treatment and care of people infected with HIV. 

Therefore, the aim of this study was to determine the variation of relative counts for CD4+ T cells using two different methodologies: (i) estimating the percentage values using a hematology counter and a flow cytometer and (ii) determination of these values only using the flow cytometer.

## 2. Methods

### 2.1. Participants and Ethics

There were 81 selected individuals with HIV. All participants were informed about the survey, and they freely signed and dated a consent form. The protocol was approved by the Ethics in Research Committee of the State University of Ponta Grossa (no. 0443710-21/2010) and was conducted in accordance with the Helsinki Declaration.

### 2.2. Laboratory Analysis

As in immunophenotyping, the determination of CD4+ T cells is the most important immunological parameter in HIV-infected individuals. The percentages of lymphocyte count obtained only by flow cytometry and the combination of the two methods (flow cytometry and hematology counter) were compared.

Biological samples were collected by a vacuum system (Vacutainer) containing the anticoagulant EDTA-K3, and two 5 mL tubes of venous blood were collected for analysis, one by flow cytometry (immunophenotyping) and one for analysis by traditional hematologic equipment (identification by impedance and roughness). All tests were performed within 6 hours of collection.

### 2.3. Percentage Values of CD4+ T Lymphocytes Estimated by Hematologic Equipment Associated with Flow Cytometry

The samples were subjected to cell count using the Cell-Dyn hematology counter 3700 (Abbott, QC, Canada) and FACSCalibur flow cytometer (Becton Dickinson-Biosciences, San Jose, CA, USA). First, we obtained the total absolute lymphocyte count using hematology equipment. Then, this absolute value of total lymphocyte was combined with the absolute values of CD4+ T lymphocytes obtained by flow cytometry in order to calculate the relative value of CD4+ T lymphocytes.

### 2.4. Percentage Values of CD4+ T Lymphocytes Obtained by Flow Cytometry

The immunophenotyping of each sample was carried out using the protocol for T-cell count of the Multitest/TruCount standard (monoclonal antibodies CD45+/CD3+/CD8+/CD4+) by FACSCalibur flow cytometer (Becton Dickinson-Biosciences, San Jose, CA, USA) to obtain the relative count of CD4+ cells.

### 2.5. Statistical Analysis

The Kolmogorov-Smirnov test was conducted to ensure normality, and the values showed normal distribution. The statistical procedures used involved a descriptive analysis (mean and standard deviation), correlation, and comparison between the two methodologies. The data were analyzed using Student's* t-*test for comparison of means between paired values. To investigate the correlations between the variables, we used the Pearson correlation. In the analysis of different methodologies, the correlation between the results was verified through the graphical representation of the *Bland-Altman* method. The level of significance adopted was *P* < 0.05. The data were processed by MedCalc statistical program.

## 3. Results and Discussion

In this study, the variability between relative counts for CD4+ T lymphocytes generated by flow cytometry and those estimated by an alternative methodology was analyzed. The estimated method necessitates the combination of results of hematologic equipment (absolute count of total lymphocytes) and cytometry flow (absolute count of CD4+ T lymphocytes).

Samples were grouped according to the absolute count of CD4+ T cells, resulting in the following stratification for the 81 samples analyzed: 18 samples with CD4+ T-cell counts below 200 cells/mL (132 ± 47 cells/mL), 34 samples between 200 and 500 cells/mL (342 ± 74 cells/mL), and 29 samples with counts above 500 cells/mL (701 ± 156 cells/mL).

The results of the percentage counts of CD4+ T cells obtained directly by flow cytometry were 10.99 ± 3.99 for stratum CD4+ < 200 cells/mL, 22.89 ± 6.47 for stratum 200–500 cells/mL, and 29.84 ± 10.46 for stratum > 500 cells/mL. However, the estimated values obtained by the hematological counter were 11.86 ± 5.10, 25.08 ± 9.07, and 36.07 ± 16.78, respectively, for each of these strata. There were identified significant differences between values for the relative counts from these two methodologies for every studied stratum (*P* < 0.05).

**Figure 1:**
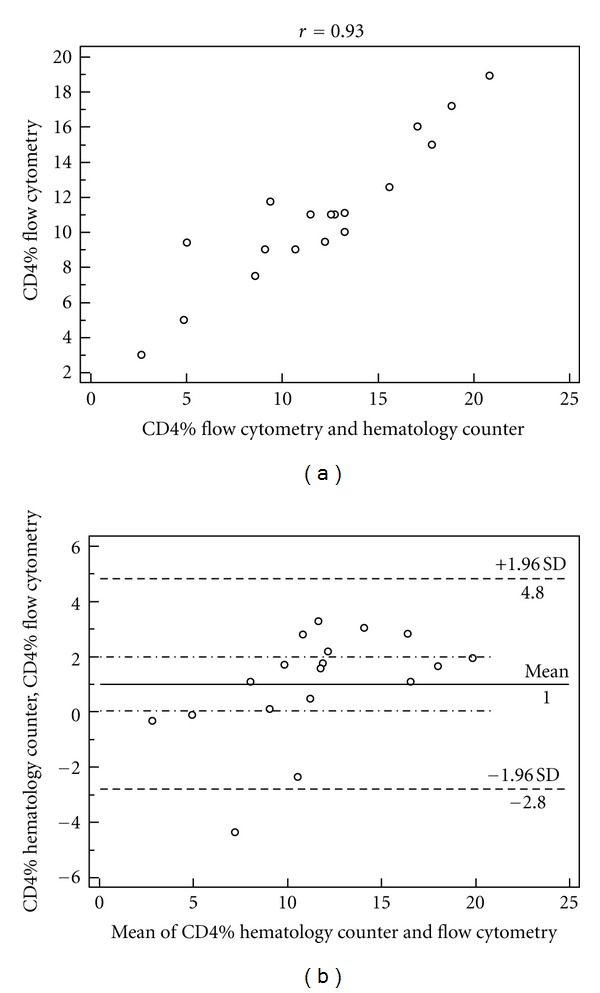
Correlation of percentage values of CD4+, *P* < 0.05 (a) and limits of agreement between the values estimated by *Bland-Altman* analysis (b) obtained by the hematology counter and the flow cytometer in the stratum of CD4 count <200 cells/mL.

**Figure 2:**
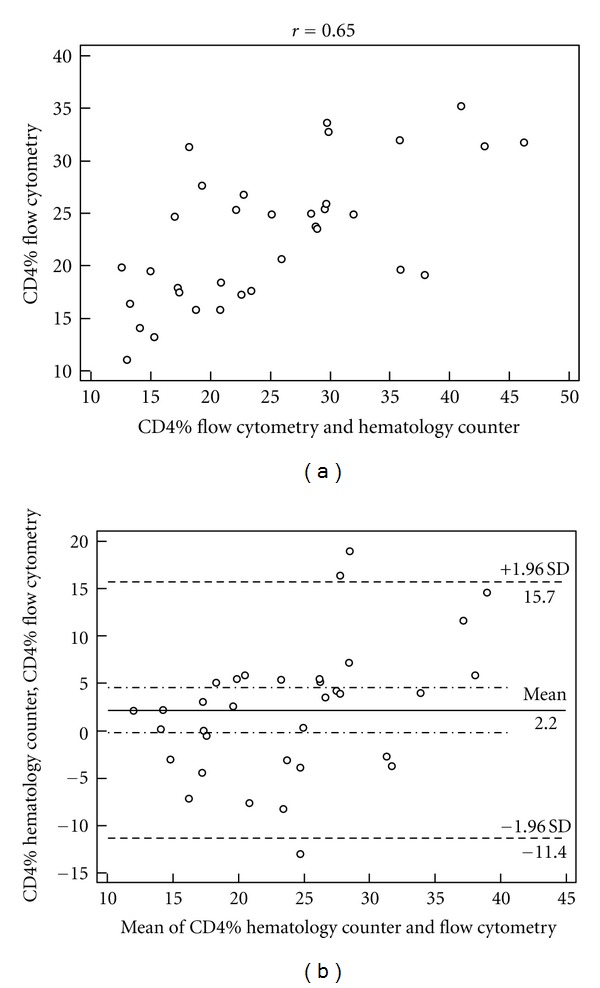
Correlation of percentage values of CD4+, *P* < 0.05 (a) and limits of agreement between the values estimated by *Bland-Altman* analysis (b) obtained by the hematology counter and the flow cytometer in the stratum of CD4 count between 200 and 500 cells/mL.

**Figure 3:**
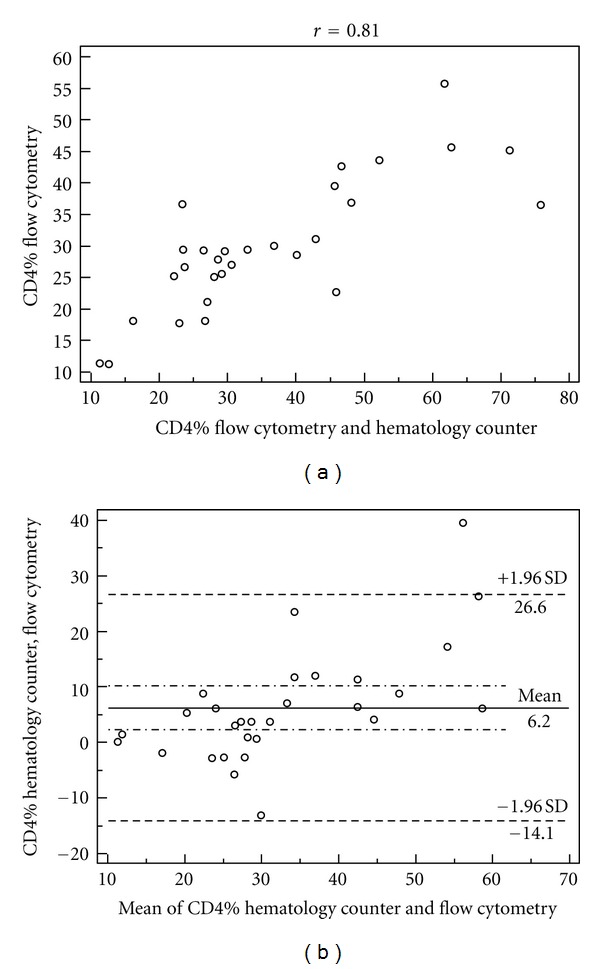
Correlation of percentage values of CD4+, *P* < 0.05 (a) and limits of agreement between the values estimated by *Bland-Altman* analysis (b) obtained by the hematology counter and the flow cytometer in the stratum of CD4 count >500 cells/mL.

The correlation between the percentages of CD4+ T lymphocytes obtained by the two methodologies for the three strata of CD4 cells studied is shown in Figures 1(a), 2(a), and 3(a), as well as the agreement represented by the *Bland-Altman* analysis shown in Figures 1(b), 2(b), and 3(b).

Studying the *Bland-Altman* analysis, it can be seen that the difference between the two measures was 1.0% for the stratum of CD4+ < 200 cells/mL, and the limits of agreement were from −2.8% to 4.8%. In the CD4 strata between 200 and 500 cells/mL, lymphocyte counts above 500 cells/mL were observed as well as broader concordance limits between 2.2% (−11.4% to 15.7%) and 6.2% (−14.1% to 26.6%), respectively, compared to the extract of CD4+ < 200 cells/mL.

It was noted that the estimate of the count of CD4+ T cells from the hematology counter was higher in relative values for the three strata studied, ranging from about 1% for the stratum CD4+ < 200 cells/mL up to 6% for the stratum > 500 cells/mL. A possible explanation for these differences is the form used for the determination of total lymphocytes by the two devices. The additional variability of the count is due to a greater inaccuracy in the way in which the hematologic equipment classifies total lymphocytes [17].

The results corroborate information reported earlier showing that the lymphocyte count obtained from hematologic analyzers is prone to errors [17–20], and, on the other hand, the use of a gate on CD45+ cells labeled with an associated dispersion parameter light using the flow cytometer provides better precision and accuracy in the quantification of lymphocytes in relation to the parameters of cell volume and conductance of hematologic counters [6].

Comparing the percentage of CD4+ T cells in the stratum of CD4+ < 200 cells/mL by the hematology counter and flow cytometry showed that the measures have a strong correlation (*r* = 0.93). However, they do not show a good agreement, since the *Bland-Altman* plot (Figure 2(a)) shows that the difference between the two methods was 1.0% of CD4+ T lymphocytes, and the limits of agreement were ±3.8%.

To the stratum of CD4+ between 200 and 500 cells/mL, it is noted that the measures are moderately correlated (*r* = 0.65). Despite the correlation, Figure 2(b) (*Bland-Altman*) demonstrates broader limits of agreement of approximately 2.2 ± 13.5%. This is similar to the lymphocyte count above 500 cells/mL with limits of agreement approximately 6.2 ± 20.4%, as shown in Figure 3(b) (*Bland-Altman*), although this stratum showed a strong correlation (*r* = 0.81).

 Similarly, MacLennan et al. [20], assessing the use of flow cytometry to provide only the absolute count of CD4+ T cells (associated with total lymphocyte count in hematology analyzers to obtain the percentage of CD4+ T lymphocytes), obtained bias of 0.92% and limits of agreement between 5.83% and 7.66% through FacsCount and Multitest/Tubs Trucount method and in FACSCalibur flow cytometer, for the absolute count of CD4+ T cells below 200 cells/mL.

The main importance of using percentage values of CD4+ T lymphocytes is in the absolute count changes in response to stimuli that are independent of HIV infection, and the percentages are less subject to this variability [13]. Considering the percentage values of CD4+ T lymphocytes for evaluation of HIV-infected individuals, the stratum CD4+ < 200 cells/mL counting could be underestimated by up to 4.8% or overestimated up to 2.8%, while for the strata 200 < CD4+ < 500 cells/mL and CD4+ cells > 500 cells/mL, the count could be underestimated by up to 15.7% and 26.6% or overestimated by up to 11.4% and 14.1%, respectively, as presented in Figures 1, 2, and 3.

## 4. Conclusions

In this study, the analysis of agreement between the hematology meter and the flow cytometer showed relatively large limits for the analyzed strata, indicating high variability.

So, although there was a good correlation between the percentage values of CD4+ T lymphocytes estimated by the two methods association, the correlation between individual measurements indicated relatively large limits for all strata of CD4+ cells studied. From a clinical standpoint, the differences given by the limits of agreement of the percentage values of CD4+ T lymphocytes could cause a conflict in decisions regarding treatment and care of people infected with HIV. Therefore, the interpretation of the percentage count of CD4+ T lymphocytes for immune monitoring of patients with human immunodeficiency virus should carefully take into account variations that may occur due to the methodology used.
